# Significance of Coronavirus Mutants in Feces and Diseased Tissues of Cats Suffering from Feline Infectious Peritonitis

**DOI:** 10.3390/v1020166

**Published:** 2009-08-26

**Authors:** Niels C. Pedersen, Hongwei Liu, Kimberly A. Dodd, Patricia A. Pesavento

**Affiliations:** 1 Center for Companion Animal Health, School of Veterinary Medicine, University of California, One Shields Avenue, Davis, CA, 95616, USA; E-mail: hwlliu@ucdavis.edu (H.L.); 2 School of Veterinary Medicine, University of California, One Shields Avenue, Davis, CA, 95616, USA; E-Mail: kadodd@ucdavis.edu (K.A.D.); 3 Department of Pathology, Microbiology and Immunology, School of Veterinary Medicine, University of California, Davis, CA, 95616, USA; E-Mail: papesavento@ucdavis.edu (P.A.P.)

**Keywords:** feline infectious peritonitis, FIPV, FECV, internal mutation, 3c gene, viral variants

## Abstract

The internal FECV→FIPV mutation theory and three of its correlates were tested in four sibs/half-sib kittens, a healthy contact cat, and in four unrelated cats that died of FIP at geographically disparate regions. Coronavirus from feces and extraintestinal FIP lesions from the same cat were always >99% related in accessory and structural gene sequences. SNPs and deletions causing a truncation of the 3c gene product were found in almost all isolates from the diseased tissues of the eight cats suffering from FIP, whereas most, but not all fecal isolates from these same cats had intact 3c genes. Other accessory and structural genes appeared normal in both fecal and lesional viruses. Deliterious mutations in the 3c gene were unique to each cat, indicating that they did not originate in one cat and were subsequently passed horizontally to the others. Compartmentalization of the parental and mutant forms was not absolute; virus of lesional type was sometimes found in feces of affected cats and virus identical to fecal type was occasionally identified in diseased tissues. Although 3c gene mutants in this study were not horizontally transmitted, the parental fecal virus was readily transmitted by contact from a cat that died of FIP to its housemate. There was a high rate of mutability in all structural and accessory genes both within and between cats, leading to minor genetic variants. More than one variant could be identified in both diseased tissues and feces of the same cat. Laboratory cats inoculated with a mixture of two closely related variants from the same FIP cat developed disease from one or the other variant, but not both. Significant genetic drift existed between isolates from geographically distinct regions of the Western US.

## 1. Introduction

Feline infectious peritonitis (FIP) was first introduced as an “important disorder of cats” by Holzworth [1] and a clinico-pathologic conference on the disease was published the following year [2]. The incidence of FIP rose progressively over the next two decades. The occurrence of FIP among all cats seen at veterinary medical teaching hospitals in the USA from 1986–1995 was 1:200 among new feline visits, 1:300 among total cat accessions, and 1% of accessions at diagnostic laboratories [3]. The incidence is several times higher among kittens and young cats originating from catteries or shelters. The disease was thought to be viral when first described but no specific etiologic agent was identified at the time [4]. Zook *et al.* [5] observed virus particles in the tissues of experimentally infected cats, however, the close similarities of FIP virus (FIPV) in tissues to members of the family Coronaviridae was noted by Ward [6]. The ability of FIPV to cause either a non-effusive (dry, parenchymatous) or effusive (wet, non-parenchymatous) form of the disease was first reported by Montali and Strandberg [7]. The close genetic relationship of FIPV to coronaviruses of dogs and swine was first recognized by Pedersen *et al.* [8]. The existence of two serotypes, feline- or canine-coronavirus like, was described in 1984 [9].

FIP was originally believed to be an uncommon clinical manifestation of a ubiquitous and largely nonpathogenic agent named feline enteric coronavirus (FECV) [10]. Subsequent studies demonstrated that the agent of FIP was distinct from FECV in disease potential but that both viruses co-existed in the same population and were antigenically identical [reviewed in 11, 12]. It was subsequently hypothesized that FIPV might be a simple mutant of FECV [13], and the two viruses were later described as biotypes of each other [14]. Animal studies, with both natural [15] and experimental [16] infection, also suggest that FIPVs arise spontaneously during the course of FECV infection. Vennema *et al.* [17] demonstrated that all major structural and accessory genes of wild type FECVs were virtually identical to FIPVs from the same or closely related cats. However, 85% of FIPVs studied had deleterious mutations in a small accessory gene called 3c. These mutations, which were either deletions or introduced stop codons, were also found to be unique to each cat.

In spite of indirect and direct supporting evidence for internal FECV→FIPV mutation, the role of FECV mutation in FIP, and especially in the 3c gene, has not been given much attention in the literature of FIP [reviewed 11]. In fact, there is a general feeling that FIPV and FECV are either the same virus, with disease being dependent on the nature of the host’s immune response [reviewed 11], or that the causative mutation is in other genes [18]. Although the precise origin of FIPVs is debated, there appears to be agreement regarding the relative cell tropisms of FECVs and FIPVs. FECVs are thought to have greater tropism for the mature apical intestinal epithelium, while FIPVs are believed to have a greater tropism for macrophages [reviewed 11]. This has led to the a strongly held belief that coronaviruses found in the feces are FECV-like, while viruses found in extra-intestinal (usually lesional) tissues are FIPV-like [19].

The purpose of this study was to repeat the original work of Vennema *et al.* [17] with a new and geographically diverse group of cats and to test the major tenant of the FECV→FIPV theory and three of its possible correlates. The major tenant of the theory assumes that functional mutations in the 3c gene are somehow related to the FIP biotype. The first correlate of this theory supposes that each FIP cat will have its own unique 3c mutant which is not transmitted cat-to-cat. The second correlate assumes compartmentalization of enteric and FIP biotypes to gut and internal tissues, respectively. The third correlate, if correct, should show FIPVs to be as geographically diverse as the FECVs from which they arise

## 2. Results and Discussion

### 2.1. Structural and accessory genes of Feline coronavirus from the feces and diseased tissues of cats with FIP are virtually identical with the exception of truncating mutations in the 3c gene

**Figure 1.**
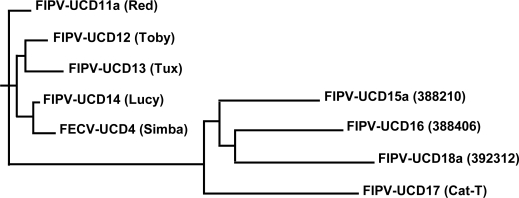
Phylogenetic analysis based on the sequences encoding for structural (E, M and N) and accessory (3a–c and 7a,b) genes. FIPV-UCD11a, 12, 13, and 14, and FECV-UCD4, were isolated from 4 related kittens and a healthy housemate. FIPV-UCD15a, 16, 17, and 18a were isolated from unrelated and geographically disparate cats.

**Figure 2.**
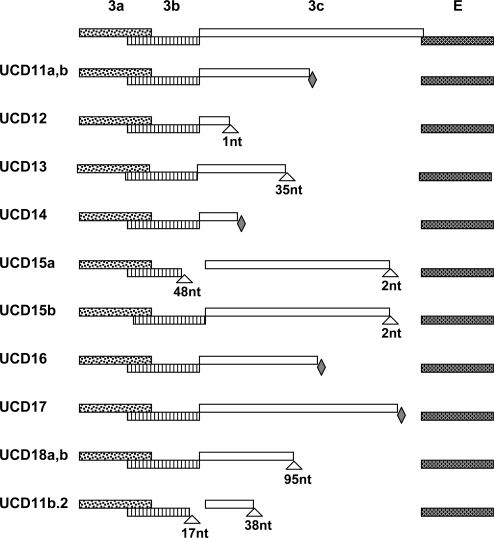
Cartoons show the 3a–c and E gene segments of FIPV-UCD11 to 18 and a cat-passaged variant of FIPV-UCD11b (FIPV11b.2). Deletions are indicated with triangles with number of nucleotides (nt) indicated; nonsense mutations are indicated with filled diamonds.

Complete structural (S, E, M, N) and accessory (3a–c and 7 a, b) gene sequences were obtained from diseased omentum of the four related cats that died of FIP and the isolates designated were FIPV-UCD11, 12, 13 and 14 (Table 1). The numbers of nucleotides sequenced for isolates FIPV-UCD11 to UCD14 are shown in Table 1, while the relationship of the FIPVs isolated from the 4 related Scottish Fold cats is shown in Figure 1. The overall sequence identity for the nine structural and accessory genes was ≥ 99% with only a small number of mutations among the four highly related viruses (Table 1). Mutations consisted of minor SNP changes, and less commonly deletions that appeared to be randomly scattered among the genes that were sequenced; about one half of the mutations resulted in amino acid changes (Table 2). Among the 9 structural and accessory genes of the four related cats, the highest genetic variability was in the 3c gene, followed by the S and M genes (Table 2). The least variability was detected in the 3b, 7a, and 3a accessory genes. Among all of the genes sequenced, only the 3c genes of the FIPV isolates had SNPs that resulted in premature stop codons or deletions that caused frame shifts; both resulting in a variable truncation of the 3c protein (Figure 2).

The omentum viruses from Red consisted of two distinct variants, as determined by sequences obtained from multiple overlapping PCR products (Table 1). These variants were designated FIPV-UCD11a and -UCD11b. There were only five SNPs scattered across the nine structural and accessory genes between the two variants. Two variants were also sequenced from the omentum of Toby, one with a non-functional 3c gene (FIPV-UCD12) and one with a functional 3c gene (FECV-UCD5). These two variants were identical in sequence except for a single-base deletion in the 3c of one of the variants (FIPV-UCD12).

Four additional unrelated cats (392312, 384062 and 388210) from Paradise, Menlo Park, and San Jose, CA, respectively, and Cat-T from Mountlake Terrace, WA were included in the study. The three cats seen at the VMTH suffered from the non-effusive form of FIP, while the Washington state cat died of effusive FIP. The E, M, N, 3a–c and 7a, b genes were amplified from the omentum or organ granulomas of all four animals. Viruses were readily detected in the diseased tissues of cats 388210, 388406, Cat-T, and 392312 and designated FIPV-UCD15 to UCD18, respectively (Table 1). FIPV-UCD15a possessed a two-nucleotide deletion near the end of the 3c gene and a second deletion of 48-nucleotide involving the terminus of 3b and beginning of 3c (Figure 2). Mutations of the 3c gene in FIPV-UCD16 and -UCD17 involved premature stop codons. Two variants with six scattered SNPs and an identical deletion in the 3c genes were identified in organ granulomas of cat 392312 and designated FIPV-UCD18a and -UCD18b (Table 1).

All of the structural and accessory genes that were sequenced for the eight different FIP cats appeared to be intact, except for the 3c genes. The 3c genes from all eight isolates contained deletions or SNPs that either produced truncating frame shifts or premature stop codons (Figure 2). The sequence relationship of the four unrelated FIPV isolates to each other and to the FIPV isolates from the four related Scottish Fold cats is shown in Figure 1. The overall genetic similarity for the E, M, N, and 3a–c, 7a, b genes ranged from 89–99% among the 8 FIPV isolates. The four FIPVs from unrelated cats showed sequence identity of 89–92% to each other and to the FIPVs from the four related cats.

Feces or colonic scrapings from the four related cats and a fifth unrelated housemate contained feline coronaviruses (Table 3). The amount of viral RNA in feces in cats with FIP was much lower than in diseased omentum and obtaining complete sequences of all 9 genes was not always possible. Therefore, the actual genes sequenced for each fecal coronavirus isolate are shown in Table 3. Coronaviruses isolated from the feces of two cats, Tux and Toby, were ≥99% identical and contained identical 3c gene mutations to the omental viruses from the same cats. The coronavirus isolated from Lucy’s feces (designated FECV-UCD3) had an intact (i.e., wild type or non-deliterious) 3c and its sequence was otherwise 99% identical to the sequence of FIPV-UCD14 found in her diseased omentum. The sequence obtained from the fecal virus of Simba, a housemate of Lucy, also had an intact 3c gene and was designated FECV-UCD4. FECV-UCD4, was most closely related to the FIPV isolated from Lucy and was 99.7% related to the consensus nucleotide sequences of coronaviruses obtained from the four related FIP cats (Figure 1, Table 2).

A similar finding was found for the cats that were unrelated to those described above and that were from disparate geographic regions. Three of four fecal samples (388210, 388406 and Cat-T) contained amplifiable RNA and complete 3c genes were sequenced in 2/3 of these cats (388210 and 388406). The 3c gene sequence of the fecal virus of cat 388406 was intact and ≥99% related to the FIPV found in diseased tissue (Table 2). This fecal isolate was designated FECV-UCD6. The 3c gene of 388210 fecal virus contained a deliterious two-nucleotide deletion near the end of the 3c gene and was designated FIPV-UCD15b. This same deletion was also detected in the lesional FIPV-UCD15a. However, FIPV-UCD15a did not contain the 48-nucleotide deletion involving 3b and 3c of FIPV-UCD15b (Figure 2). Only the 7a, b genes were sequenced from the feces of Cat-T and the sequence was 100% identical to the 7a, b sequence from the omental FIPV-UCD17.

This study of lesional and/or fecal coronaviruses from nine cats both supported and modified the previous conclusions of Vennema *et al.* [17]. Viruses from diseased tissues from all eight cats in this study had truncating mutations, either in the form of deletions leading to frame shifts or coding changes causing premature stop codons in the 3c gene. Such damaging mutations were not present in other accessory and structural genes in this or in a previous study [17]. As with the earlier study [17], all or almost all of the fecal isolates from diseased cats and a healthy contact control animal had intact 3c genes. Taken as a whole, the present study supported a role for deleterious 3c gene mutations in the genesis of FIPVs from FECVs. However, not all FIPV isolates have deleterious 3c gene mutations. Although 8/8 (100%) of lesional isolates in the present study had functional mutations in their 3c genes, only 11/13 (85%) of the FIPVs reported by Vennema *et al.* [17] had deliterious 3c gene mutations. We have also recently observed what appeared to be intact 3c genes in 12/31 random breed cats that were adopted from a large shelter in Northern California and died of FIP. However, several of these isolates contained mutated 3c genes as minor variants, and without animal inoculation studies it is not possible to say whether or not these or the remaining isolates were capable of causing FIP. The existence of helper/defective virus replication in the latter situation also needs to be considered. Animal inoculation studies to determine the biotype of a given feline coronavirus are critical for determining the ultimate biotype of any isolate, regardless of its sequence regularities or irregularities. It was therefore important to demonstrate herein that an isolate from the four related cats reported herein was capable of causing FIP.

Since some FIPVs appear to have intact 3c genes, it may be premature to ascribe the FIP biotype solely to deleterious mutations in the 3c gene. However, what are the alternatives? It can be argued that mutations in the conserved replicase/transcriptase genes may have a similar effect; that small mutations in other structural and accessory genes, collectively or singly, will have the same effect; that FIPV and FECV are identical viruses; or that deleterious 3c gene mutations are an effect of the disease and not its cause. Involvement of the replicase/transcriptase genes is unlikely, because the replicase/transcriptase region is highly conserved among feline coronaviruses and unlikely to be involved in cell tropism or evasion of the host’s immune response. One study of a natural serotype I FIPV isolate (C1Je) showed a high degree of sequence conservation within the replicase/transcriptase genes compared to other feline coronaviruses, while a premature stop codon limited the 3c gene product to the first 16 amino acids [19]. It is also unlikely that mutations in other accessory or structural genes are involved, even though such mutations have been frequently found in feline coronaviruses. Firstly, 3c gene mutations in FIPVs occur significantly out of proportion to mutations in other structural or accessory genes. Secondly, there is little scientific evidence, especially based on animal inoculation, that other accessory genes are involved in FIP. In the original report that proposed the internal mutation theory, 11/13 of the FIPVs had 3c mutations, while 2/13 isolates had only 7b mutations [17]. However, both of the latter cats were related and had been experimentally infected with an identical FECV (FECV-RM); a third sibling cat from this group had the same 7b mutation but with a unique functional 3c mutation. Variants were not tested at the time and it is possible that 3c mutants would have been present if the two discordant isolates had been adequately sequenced. Earlier studies have also demonstrated an absence of 7b mutations in almost all FECVs and other FIPVs and indicate that such mutations are most likely tissue culture artifacts [17, 20]. Yet other studies suggest that 7a and 7b mutations occur in nature in both FIP and enteric infections and are therefore not directly linked to pathogenicity [20, 21, 23].

There is a general belief that host and environmental factors, and not virus mutation, are the basic determinants of whether a cat develops FIP or just a mild enteritis following exposure to the common feline coronavirus [24–28]. For such a theory to be correct, FECVs and FIPVs would have to be identical in both genetic structure and virulence. The evidence that FECVs and FIPVs cause very different diseases is strong [10–13]. Even though environmental and host factors are admittedly important in FIP [29, 30], lesional viruses from the eight FIP cats in this study, even though highly related to fecal isolates, were easily differentiated from each other based on deliterious 3c gene mutations alone. Moreover, an infectious inoculum made from the diseased omentum of one of the FIP cats induced FIP in 3 of 12 cats that were experimentally infected (see section 2.2.). Confirmation of biotype by animal inoculation, such as described herein, is rarely done in published reports concerned with feline coronavirus infection [reviewed in 11].

The possibility that deleterious 3c gene mutations are an effect of the disease and not a cause also has to be considered. However, there is little precedence for this and given the ability of a lesional isolate from the present study to produce FIP, it is counterintuitive for a functional 3c gene mutant to be both a cause and effect of its own disease. This theory would also not explain why all non-tissue culture adapted FECV strains used for experimental inoculation studies have intact 3c genes, while all tissue culture adapted and non-adapted strains have mutated 3c genes [17].

The existence of feline coronavirus variants was not a novel observation [23, 31, 32], but their frequency and fate has not been previously addressed. Variant forms were found in both extraintestinal tissues and feces of the 9 cats in this study, but only one variant became predominant upon experimental passage from one cat to another (see section 2.2). The infecting variant may have been merely the first virus into a macrophage, or its selection may have involved more complex host/virus interactions. We also found that subtle, and sometimes significant, genetic mutations (usually SNPs and deletions) occurred upon primary replication in a new host. Therefore, genetic variation among feline coronaviruses occurs both within and between host cats. Selective infection with a single variant can also rapidly lead to genetically distinct clades of coronavirus, especially when combined with a high intrinsic and extrinsic mutation rate.

### 2.2. Experimental infection of laboratory cats establishes that FIPV-UCD11a, b possess the FIP biotype and that co-infection with both variants leads to infection with one or the other variant but not both

Twelve laboratory cats were inoculated intraperitoneally with a cell-free inoculum prepared from the diseased omentum of Red, which contained two variant forms of the virus (FIPV-UCD11a and - UCD11b). Three of these cats developed effusive FIP within 2–4 weeks. Viral RNA was isolated from the omentum of each experimentally infected cat at the time of necropsy. The S (one cat) and E, M, N and 3a–c, 7a, b genes (all three cats) were sequenced. One of the cats was found to be infected with FIPV-UCD11a, while two of the cats were each infected with FIPV-UCD11b. Each of these cats had a nearly identical variant of UCD-11a or UCD-11b in its diseased omentum (Table 4). The premature stop codon of parental 3c gene was preserved in FIPV isolates from all three cats. However, FIPV-UCD11b. 2 isolated from one of the three cats had acquired two additional large deletions affecting both the 3b and 3c genes that were not in the infecting virus (Table 4 and Figure 2).

It is important to determine by animal inoculation studies the true biotype of a feline coronavirus that is being reported, rather than always referring to a generic feline coronavirus [reviewed in 11]. Feline coronaviruses that possess the FIP biotype, such as FIPV-UCD11a,b, will readily induce FIP in from 25–100% of infected individuals, depending on the strain being tested [reviewed in 11]. However, bonifed (cat-to-cat passaged, non-tissue culture adapted) FECV strains will rarely induce FIP in healthy cats [9, 12, 15, 17].

### 2.3. Significant sequence differences exist between feline coronavirus isolates from disparate geographic regions of the Western US

The present study adds to our knowledge of genetic drift among feline coronaviruses that inhabit the same cat, multi-cat household, cattery, or geographically distant region. All of the FIPVs and FECVs isolated from the five cats that had close contact with each other in Sonoma, California were ≥99% related (Tables 1 and 3; Figure 1). Based on gene sequences and historical facts, it can be reasonably concluded that cat Simba was infected with an FECV following contact with cat Lucy. This supported another correlate of the internal mutation theory; FECVs are easily spread cat-to-cat, while FIPVs are not. Addie *et al.* [33] also noted that the same strain of coronavirus tended to persist among any given group of cats. However, coronaviruses within a closely housed group of cats, and even within the same cat, undergo continuous genetic drift. We observed sequence differences of 1–2% or less in cats from the same group, while genetic drift between cats from distant areas of the western US was on the order of 6–16%. Herewegh *et al.* [34] also found that feline coronaviruses from individuals within the same environment had unique genetic fingerprints and fell within the same clade, while geographically distant isolates belonged to genetically unique clades. The notable mutational drift observed among feline coronaviruses across geographic regions, in the face of genetic conservation within stable groups of cats, is paradoxical. However, the evidence indicates that coronavirus infection in any group of cats originates from a single founder virus, that virtually every cat in a group is infected rapidly and efficiently, and that cats appear to resist superinfection with closely related strains [34]. The single founder virus effect was confirmed in the present study (Table 4). Thus, marked genetic drift occurs when a single coronavirus strain is serially passed from one susceptible population to the next. This scenario was supported by our animal transmission studies; when cats were simultaneously infected with two closely related variants of FIPV, each variant segregated into different cats. Therefore, minor mutants may become predominate when passed cat-to-cat.

### 2.4. All of the feline coronavirus isolates in this study were serotype I as based on both S and 3a gene sequences

**Figure 3.**
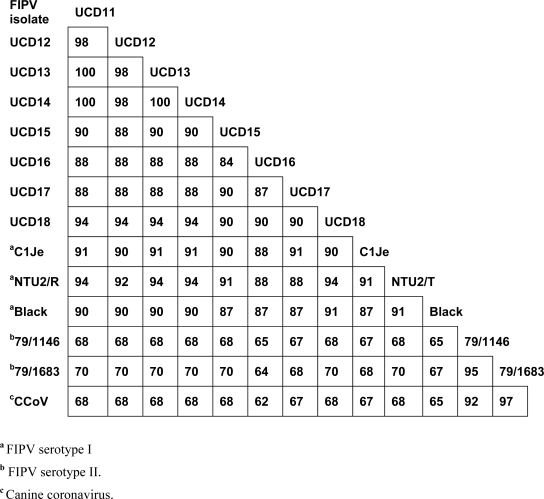
A comparison of the amino acid sequences of 3a proteins from several different FIPVs and a canine coronavirus (CCoV). The reference sequence is from FIPV-UCD11. GenBank accession numbers for additional FIPV isolates are EU186072(Black), DQ160294(NTU2/R), DQ848678(C1Je), NC_007025(WSU-79/1146), X80799(WSU-79/1683), and DQ112226(CCoV).

The S sequences of FIPV-UCD11 to 14 were compared to that of previously reported FIPVs and to a purported FECV (WSU-79-1683) (data not shown). The S protein shared 98% sequence identity among the four FIPV isolates, and 87–91% sequence identity to other published serotype I FIPVs. However, when compared to the S protein of serotype II feline coronaviruses WSU-79-1146 and WSU-79-1683, there was only 43–44% sequence identity (data not shown). Based on the comparison of S proteins, FIPV-UCD11 to 14 were classified as serotype I feline coronaviruses. However, these studies demonstrate that serotype designation of feline coronaviruses can be more easily made from comparisons of the much smaller 3a rather than significantly larger S sequences (71 vs 1471 amino acids in the respective gene products) (Figure 3). Similar to the S protein comparison, the 3a sequence of all four FIPVs from the related cats shared 98–100% sequence identity to each other, and 84–94% sequence identity to the four FIPVs from unrelated cats and from published serotype I FIPVs (Figure 3). However, when compared to the 3a protein of serotype II feline coronaviruses WSU-79-1146 and WSU-79-1683 or to 3a of Canine coronavirus, there was only 65–70% sequence identity. These results indicate all eight FIPVs from this study clearly belonged to serotype I based on their 3a protein sequences, while known serotype II viruses and the canine coronavirus formed a separate group (Figure 3).

### 2.5. There was no evidence for cat-to-cat (i.e., horizontal) transmission of 3c gene mutants among cats in the same environment

FIPV is unique from most other viruses, because it is infrequently spread from animal-to-animal in a horizontal manner, yet it is highly infectious when extracts of diseased tissues or fluids are inoculated into naïve cats by a number of routes [reviewed in 11]. The general belief is that enteric biotypes are compartmentalized to the gut, while FIP biotypes are found only within internal organs [19]. However, viruses with 3c mutations identical to FIPVs from lesional tissues were present in the feces of some cats in this study (Table 3), thus making horizontal transmission theoretically possible in certain circumstances. There is also evidence that FIPV may have been shed in urine of FIPV infected cats [35], and that coronavirus may be present in the blood, especially among younger cats [36]. There are also several reports of FIP outbreaks of sufficient magnitude and acuteness to suggest horizontal transmission [reviewed in 11]. While this study did not answer the question as to the relative importance of vertical and horizontal transmission, it indicated the need to carefully study fecal and lesional virus isolates that are involved in explosive, large scale, epizootics of FIP and not just the common enzootic form.

### 2.6. Precedence and possible role for functional 3c gene mutations

Positive proof that the 3c protein is responsible for the FIP phenotype, in at least a proportion of cats dying of FIP, will require knowledge of its exact function, of which we currently know very little. A GenBlank blast search shows a 30% genetic homology between feline coronavirus 3c and SARS coronavirus 3a (data not shown). Moreover, the 3c protein of feline coronavirus also has an identical hydrophillicity profile to its own M protein and to the M and 3a proteins of SARS coronavirus [37]. These similarities prompted Oostra and colleagues [37] to state – “(…) it appears that all group 1 [corona] viruses expresss group-specific proteins predicted to be triple-spanning membrane proteins. Examples are the feline ORF 3c protein and the HCOV-NL63 ORF 3a protein (…) Despite the small amount of sequence homology among this protein, the similarities in their hydropathy profiles, both to each other and to the corresponding M proteins, as well as to the SARS-CoB 3a protein, are quite remarkable. Nothing is known about these proteins, but it is clear that it will be interesting to learn more about their biological features.” A great deal of research has been reported, and is being conducted, on the SARS coronavirus 3a gene and protein and it is evident that this gene and its product play an important role in viral assembly, spread and pathogenesis, as well as to protective immunity [38–40]. If the 3c protein of feline coronavirus truly has an analogous function to SARS coronavirus 3a protein, SARS coronavirus research might be applicable to feline coronaviruses and how they cause disease.

## 3. Experimental Section

### 3.1. Feline coronavirus terminology

The authors have used the original names of FECV to refer the enteric biotype of feline coronavirus, and FIPV for the FIP biotype. Published non-tissue culture adapted coronavirus isolates from the feces of healthy cats always possess an intact or wildtype 3c, while strains from FIP diseased tissues have mutated 3c genes [17]. Therefore, the designation of FECV or FIPV in this study was applied to isolates with 3c genes that yielded either intact or truncated proteins, respectively. The generic term “coronavirus” or “feline coronavirus” was used herein when not referring to a specific biotype.

### 3.2. Subjects

Four Scottish Fold kittens were born into the same cattery in Sonoma, California; Red, Toby and Lucy were from the same litter of three, while Tux was born a week later in a litter of three to a sister queen and the same tom. Simba, an 11 year-old American curl, was born in an unrelated cattery and resided in another Sonoma household as a pet. Lucy was placed into this household with Simba when she was 17 weeks old, while Red went to live in another home with two other older cats when at 14 weeks of age. Tux and Toby remained in their home cattery with several other cats. Lucy, Tux, Red and Toby first showed signs of indicative FIP at 23, 33, 35 and 40, weeks of age, and were euthanatized with confirmed disease at 27, 37, 39 and 41 weeks of age, respectively. All other contact cats have remained healthy to this time.

Four additional cats were recruited from the western US. Two of them were 26- and 60-month old Burmese (388406 and 392312) from Paradise and Menlo Park, CA, respectively. The third was a 16-month old Birman (388210) from San Jose, CA, and the fourth was a 2-year old Sphinx (Cat-T) from Mountlake Terrace, WA (courtesy Dr. Tracy Tomlinson). Full necropsies on all cats, except Cat-T were performed at the School of Veterinary Medicine Teaching Hospital (VMTH), University of California, Davis, CA. Cat-T was necropsied at a private veterinary diagnostic laboratory (Phoenix Central Laboratory, Everett, WA).

A definitive diagnosis of FIP was confirmed on all eight cats by gross and microscopic examination of tissues and immunohistochemistry. The four related Scottish Folds and Cat-T suffered from the effusive form of FIP, while the two Burmese and one Birman cats suffered from non-effusive FIP. Samples of diseased omentum (effusive FIP) or kidney granulomas (non-effusive FIP), along with feces (or colonic mucus/mucosal scrapings from one cat) were collected at the time of necropsy and stored at −20°C. Feces from the healthy sentinel cat, Simba, were also collected.

### 3.3. FIPV transmission studies

A cell free inoculum was made from the diseased omentum of Red, one of the four related cats. Omentum was frozen in liquid nitrogen and ground to a powder. The frozen omental powder was reconstituted in 0.25g/ml HBSS (Hanks buffered saline solution) and centrifuged twice at 2,000 × g for 30 minutes. The supernatant was stored at −70°C as viral stock. The viral stock was diluted 1:3 with HBSS when used as inoculum for the FIPV transmission study. Adult specific pathogen free cats were obtained from the breeding colony of the Feline Health and Pet Care Center, School of Veterinary Medicine, University of California, Davis, CA. A total of twelve cats were inoculated intraperitoneally with 1 ml of cell free viral inoculum. Three cats developed FIP within 2-4 weeks and complete necropsies established that all three cats had effusive FIP. Diseased tissues and feces were collected for isolation of feline coronavirus RNA.

### 3.4. Isolation of viral RNA

Viral RNA was extracted from omentum, granulomas of kidney, and colonic mucus/mucosal scrapings using QIAgen RAeasy mini kit (QIAgen, USA). About 30 mg ground lesional tissues were lysed with 600 μl lysis buffer containing b-mercaptoethanol. After thoroughly mixing, the lysate was homogenized with QIAshredder (QIAgen, USA) and an equal volume of 70% ethanol was added to the homogenized lysate. The lysate mixture was applied to RNeasy spin column and the RNA binding to the column was achieved by centrifugation. The RNeasy spin column was then washed and the RNA was eluted with 50 μl RNase-free water and stored at −70°C.

Feces from 8/9 cats were suspended with 5 volumes of phosphate buffer saline (PBS) by vortexing. The suspension was centrifuged at 8,667 × g for 10 min and the supernatant transferred to a new tube and centrifuged at 54,174 × g for 30 min. The pellet containing the virus was suspended with 5 ml PBS and centrifuged again at 54,174 × g for 30 min. The pellet was suspended in 140 μl PBS and the viral RNA extracted using a QIAamp Viral RNA mini kit (QIAgen, USA). Briefly, 560 μl lysis buffer containing carrier RNA was mixed with the 140 μl viral suspension and incubated at ambient temperature for 10 min; 560 μl 100% ethanol was added to the lysate. The lysate mixture was applied to QIAamp mini spin column and the RNA binding to the column was achieved by centrifugation. The column was then washed and the RNA was eluted with 50 ml RNase-free water and stored at −70°C.

### 3.5. cDNA synthesis and PCR amplification of viral sequence

**Figure 4.**
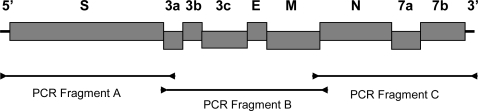
A cartoon shows the structural (S, E, M, and N) and accessory (3a, 3b, 3c, 7a, and 7b) genes of feline coronavirus. The PCR primers used for PCR amplifications are shown as arrows. The PCR products are represented by a black line joining the forward and reverse PCR primers.

The published sequences of feline coronaviruses in GenBank were used to design the primers for a reverse transcripase polymerase chain reaction (RT-PCR). Three primer pairs were designed from highly conserved regions and used to amplify three overlapping fragments containing the nine structural and accessory genes of feline coronavirus (Figure 4 and Table 5). The RT-PCR was carried out with QIAgen LongRange 2Step RT-PCR kit (QIAgen, USA). The viral RNA was first denatured by incubating at 65°C for 5 min and then chilled on ice. The reverse transcription was carried out in 20 μl reaction mixture containing 10 units of LongRange reverse transcriptase, 0.8 unit of RNase inhibitor, 1 mM dNTP, 1 mM Oligo dT, and 5 μl of denatured viral RNA in 1× reaction buffer. The mixture was incubated at 42°C for 2 hr followed by 85°C for 5 min. The reverse transcribed cDNA was stored at −20°C or used immediately in PCR amplification. The viral cDNA was amplified in 20 μl reaction mixture containing 2 μl cDNA, 1 unit LongRange PCR enzyme mix, 0.5 mM dNTP, 0.25 mM forward primer, and 0.25 mM reverse primer in 1× PCR buffer. The mixture was then incubated at 93°C for 3 min and amplified for 30 cycles at 93°C for 30 s, 60°C for 30 s, and 68°C for 1 min per kb of PCR product, followed by a final extension for 10 min at 68°C. The reverse transcribed viral RNA from feces was amplified for 40 cycles under the same condition. The PCR products were electrophoresed in TAE buffer on a 0.8% agarose gel. The PCR product was purified using a QIAgen gel purification kit (QIAgen, USA).

### 3.6. Cycle sequencing

Sixty primers were ultimately used for sequencing, with the S gene requiring the most primer development and modification (primer sequences not shown). Regions containing mixed sequences due to the presence of a minor variant were also resolved with overlapping primers. The purified overlapping PCR products encoding the nine structural and accessory genes were sequenced with a BigDye Terminator v3.1 Cycle Sequencing Kit (Applied Biosystems, USA) in 15 μl reaction containing 1 μl Big Dye terminator mix, 2 μl reaction buffer (5×), 35 ng sequencing primer, and 3 μl (out of 50 μl) gel purified PCR product. The sequencing reaction was incubated at 93°C for 2 min and then amplified for 40 cycles at 93°C for 20 s, 50°C for 20 s, and 60°C for 4 min. Unincorporated dye terminators and dNTP were removed by gel filtration based Performa DTR Ultra 96-well plate kit (EdgeBio, USA) and the cycle amplified products were analyzed by capillary electrophoresis using an ABI 3730 Genetic Analyser (Applied Biosystems, USA). Vector NTI advance 10 software (Invitrogen, USA) was used for alignment of sequence data. The percent sequence identity for pairwise alignment and the phylogenetic relationship among different FIPV isolates was analyzed using ClustalW2II (www.ebi.ac.uk/tools/clustalw2/).

Tables 2 and 3 list the various FIPV and FECV isolates that were studied and the genes that were sequenced for each. These sequences have been deposited in the database of GenBank.

## 4. Conclusions

The ubiquitous form of feline coronavirus is readily passed cat-to-cat by the fecal-oral route and is the cause of a mild or unapparent enteritis. Like coronaviruses in general, feline enteric coronavirus (FECV) is undergoing constant mutation within its accessory and structural genes. Feline infectious peritonitis (FIP), which is a highly fatal systemic disease, is a sequel of FECV infection in a small proportion of cats. The virus isolated from the diseased tissues of cats with FIP is highly related to the FECV identified in the feces. Although SNP and deletion mutations were common between isolates from the same cat, only the 3c gene was rendered non functional by such mutations. Deleterious mutations in 3c tend to be found in diseased internal tissues, while viruses with intact 3c are found mainly in the feces. While deleterious mutations of the 3c gene were seen in all 8 FIP cats in this study, and in virtually all previously reported FIPVs, they are by no means a universal finding. However, there is compelling evidence that when they do occur, they are the cause of FIP and not an effect of the disease. Deleterious 3c gene mutants will readily cause FIP when inoculated into laboratory cats, whereas their largely non-pathogenic fecal counterparts with intact 3c genes are readily transmitted from one cat to another. These gene 3c mutants, when they occur, are unique to each cat with FIP, indicating that they arise independently in each host and not by mutation in one cat with subsequent horizontal spread to others. Several minor variants can co-exist in both tissues and feces and when two variants are inoculated together into the same cat, one or the other, but not both, will predominate. The high mutability of feline coronaviruses leads to minor genetic differences between cats in one closely contained geographic area, while significant genetic differences are seen between isolates from geographically disparate regions. More in depth studies on the function of the feline coronavirus 3c gene will be important for determining its precise role in FIP.

## Figures and Tables

**Table 1.** Name and biotype designation of coronavirus isolates (including variants) from diseased tissues of eight cats dying of FIP. The genes that were sequenced, their mutability, degree of relatedness, and the nature of the functional mutation in the 3c gene are given for each cat.

**Cat name**	**Isolate**	**Genes sequenced**	**# SNPs/nts sequenced^a^**	**% Sequence identity^b^**	**Status of 3c**	**GenBank Accession #**
Red	FIPV-UCD11a	S,E,M,N,3a–c,7a,b	36/8959	99.59	Early stop	FJ917519
	FIPV-UCD11b		41/8959	99.54		FJ917520
Toby	FIPV-UCD12	S,E,M,N,3a–c,7a,b	51/8984	99.43	Deletion	FJ917521
	FECV-UCD5		50/8984	99.43	Intact	FJ917522
Tux	FIPV-UCD-13	S,E,M,N,3a–c,7a,b	35/8950	99.61	Deletion	FJ917523
Lucy	FIPV-UCD-14	S,E,M,N,3a–c,7a,b	25/8958	99.72	Early stop	FJ917524
388210	FIPV-UCD-15a	E,M,N,3a–c,7a,b	na**^c^** /4608	92	Deletions in 3b–c and early stop	FJ917525
388406	FIPV-UCD-16	E,M,N,3a–c,7a,b	na/4635	92	Early stop	FJ917526
Cat-T	FIPV-UCD-17	E,M,N,3a–c,7a,b	na/4660	92	Early stop	FJ917527
392312	FIPV-UCD-18a	E,M,N,3a–c,7a,b	na/4529	91	Deletions	FJ917528
	FIPV-UCD-18b					FJ917529

a SNP differences between tissue virus and the consensus sequences of the FIPVs from the four related cats.

b The percentage sequence identity was determined by comparison to the consensus sequences of the FIPVs of the four related Scottish Fold cats.

c na - not applicable; sequence difference >6–12%

**Table 2.** Mutational variations in nucleotide and resulting amino acid sequences among the structural and accessory genes of FIPV isolates from four closely related cats involved in the same FIP outbreak.

**Isolate**	**S 4413nt/1471aa**	**3a 213nt/71aa**	**3b 221nt/74aa**	3c* 714nt	**E 249nt/83aa**	**M 796nt/265aa**	**N 1128nt/376aa**	**7a 306nt/102aa**	**7b 621nt/207aa**
**UCD11a**	19/12	0/0	0/0	6nt	3/2	4/1	1/1	0/0	3/2
**UCD12**	24/15	1/1	0/0	7nt	1/0	8/5	7/4	1/1	2/1
**UCD13**	23/10	0/0	0/0	4nt	1/1	4/4	3/2	0/0	0/0
**UCD14**	12/5	0/0	0/0	3nt	0/0	2/1	2/2	0/0	2/1

* None of the 3c genes of these FIPV isolates encoded a functional protein.

**Table 3.** Name and biotype designation of coronavirus isolates (including variants) from the feces of eight cats dying of FIP. The genes that were sequenced, their mutability, degree of relatedness to lesional isolates, and the nature of the functional mutation in the 3c gene are given for each cat.

**Cat Name**	**Isolate**	**Genes sequenced**	**#SNPs/nts sequenced^a^**	**% sequence identity**	**Status of 3c**	**GenBank Accession #**
Lucy	FECV-UCD3a	E,M,N,3a–c,7a,b	47/4554	98.97	Intact	FJ9943761
	FECV-UCD3b	E,M, 3a–c	53/2517	97.89	Intact	FJ9943762
Simba	FECV-UCD4	E, M, N, 3c, 7a,b	15/4166^b^	99.65	Intact	FJ9943763
Toby	FIPV-UCD12	3a,b,7a,b	0/2954	100	Deletion	FJ9943765 FJ9943766
Tux	FIPV-UCD13^c^	E,M,N,3a–c,7a,b	7/4486	99.84	Deletion	FJ9943764
Red	Unnamed^d^	3a,b,7b	7/1516	99.54	Unknown	FJ9943767 FJ9943768
388210	FIPV-UCD15b	3a–c,7a,b	14/2498	99.44	Deletion	FJ9943769 FJ9943770
388406	FECV-UCD6	E,M, 3a–c,7a,b	44/3595	98.88	Intact	FJ9943771 FJ9943772
Cat-T	Unnamed^d^	7a,b	0/1177	100	Unknown	FJ9943773

a # SNP differences between fecal and diseased tissue isolates from the same cat

b # SNP differences between fecal virus and consensus sequence of FECVs from other related cats.

c There were 7 SNPs between FIPVs found in colonic scraping and the diseased omentum.

d The biotypes of the virus isolated from the feces of these cats were not determined due to an inability to amplify the 3c gene.

**Table 4.** Name and biotype designation of coronavirus isolates from three cats dying of experimentally induced FIP. The genes that were sequenced, their mutability, degree of relatedness to the consensus sequence of FIPV-UCD11a, b, and nature of the functional mutation in the 3c gene are given for each cat.

**Cat #**	**Isolate**	**Genes sequenced**	**#SNPs/nts sequenced^a^**	**Type of mutation in 3c**	**GenBank Accession #**
07–036	FIPV-UCD11a.1a^b^	E,M,N,3a–c,7a,b	5/6711	Stop codon same as FIPV-UCD11a	FJ917530
	FIPV-UCD11a.1b^c^		7/6711		FJ917531
05–243	FIPV-UCD11b.1a^b^	E,M,N,3a–c,7a,b	6/4680	Stop codon same as FIPV-UCD11b	FJ917532
	FIPV-UCD11b.1b^c^		7/4680		FJ917533
98–272	FIPV-UCD11b.2a^b^	S,E,M,N,3a–c,7a,b	5/8943	Stop codon same as FIPV-UCD11b, plus deletions in *3b,c*	FJ917534
	FIPV-UCD11b.2b^c^		8/8943		FJ917535

a # SNPs detected when compared to their parental FIPV-UCD11a or FIPV-UCD11b viruses.

b Variant strain isolated from experimentally infected cat

c Variant strain used for SNP comparison

**Table 5.** Primer sequences used for the amplification of accessory and structural genes of feline coronavirus isolates.

**Primer name**	**Nucleotide sequence**	**Genome Position^a^**
Forward primer for PCR fragment A	5′TCGTTGGATTACTAAGGAAGG-3′	20121
Reverse primer for PCR fragment A	5′CTGTGATATTAAACAGTCGTATG-3′	24854
Forward primer for PCR fragment A	5′GGCCTTGGTATGTGTGGCTAC-3′	24380
Reverse primer for PCR fragment B	5′CTATTCCAATAACCAATTTGTTGATC-3′	26996
Forward primer for PCR fragment C	5′GCTACACCTAGTAGAACCATCG-3′	26605
Reverse primer for PCR fragment C	5′GTGTATCACTATCAAAAGGAAAATTTTC-3′	29125

a defined by the genome of FIPV-WSU-79/1146 (NC_0007025).
